# The metastasis associated protein S100A4: role in tumour progression and metastasis

**DOI:** 10.1038/sj.bjc.6602613

**Published:** 2005-05-17

**Authors:** D M Helfman, E J Kim, E Lukanidin, M Grigorian

**Affiliations:** 1 Department of Cell Biology and Anatomy, Sylvester Comprehensive Cancer Center, University of Miami School of Medicine, Miami, FL, 33136, USA; 2 Department of Genetics, Stony Brook University, Stony Brook, NY, USA; 3 Department of Molecular Cancer Biology, Institute of Cancer Biology, Danish Cancer Society, Strandboulevarden 49, DK-2100 Copenhagen, Denmark

**Keywords:** breast cancer, prognosis, motility, invasion, apoptosis, angiogenesis

## Abstract

The metastasis associated protein S100A4 is a small calcium binding protein that is associated with metastatic tumors and appears to be a molecular marker for clinical prognosis. Below we discuss its biochemical properties and possible cellular functions in metastasis including cell motility, invasion, apoptosis, angiogenesis and differentiation.

## S100A4 AND CANCER PROGNOSIS

S100A4 (also known as mts1 pEL-98, 18A2, p9Ka, CAPL, calvasculin, Fspl) is a member of the S100 family of calcium binding proteins and has been categorized as a metastasis-associated protein. S100A4 is a candidate as a molecular marker for metastatic potential with high prognostic significance. Support for S100A4 as a prognostic marker was first obtained from two studies of patients with early stages of breast cancer. Rudland *et al* (2000) reported a significant difference in the 19-year survival rate for patients with invasive but operable stage I and stage II breast cancer depending on the S100A4 status of their tumours (80% for S100A4-negative *vs* 11% for S100A4-positive, *P*<0.0001). The median survival of the S100A4-negative group was >228 months compared to 47 months for the S100A4-positive group. Subsequent studies confirmed S100A4 as a potential predictor for metastasis and mortality in early-stage breast cancer. Patients with S100A4-positive early tumours had a significantly worse 10-year survival than those with S100A4-negative tumors (29.0 *vs* 68.9%, *P*=0.001) (Lee *et al*, 2004). In fact, S100A4 was shown to be a significant marker of prognosis even for T1N0M0 breast cancers. This suggests that critical changes in metastatic potential may be determined early in breast cancer disease using markers such as S100A4.

In contrast to these data, Pedersen *et al* (2002) did not find a significant correlation between S100A4 expression and clinical outcome. Studies on 62 breast carcinomas of I–IV stages revealed significant correlation of S100A4 immunoreactivily with histological grade and loss of oestrogen receptor but not with the development of distant metastasis and patient survival. While these results are different from those obtained by others (Ruland *et al* and Lee *et al*), the discrepancy may be explained by fewer case numbers, inclusion of all stages of disease, a relatively short period of follow-up, and differences in sample storage conditions or fixation methods. Nevertheless, the putative role of S100A4 protein as a prognostic factor remains uncertain at this time. To further clarify the prognostic significance of this protein, additional studies of large number of cancer specimens and lengthy follow-up are needed.

In addition to these data for breast cancer, an increase in S100A4 protein expression has been correlated with a worse prognosis for patients with colorectal, gallbladder, bladder, esophageal, non-small-cell lung, gastric, medulloblastoma, pancreatic and hepatocellular cancers (Rudland *et al*, 2000; Mazzucchelli, 2002; Cho *et al*, 2003; Heman *et al*, 2003; Cui *et al*, 2004; Missiaglia *et al*, 2004). Since tumour size and histological grade do not sufficiently predict metastatic potential, there is a need to identify markers to devise treatment algorithms that guide physicians regarding which patients should receive adjuvant chemotherapy treatment. Markers such as S100A4 may help limit the use of chemotherapy with all its known toxic side effects to those patients who will most benefit.

## S100A4 EXPRESSION IS STRONGLY CORRELATED WITH AN AGGRESSIVELY METASTATIC PHENOTYPE

Ebralidze *et al* (1989) first introduced the association of S100A4 (mts 1) with metastatic capacity of tumour cells in their report that S100A4 mRNA expression level matched the metastatic potential of several tumour cell lines. This was followed by reports that transfection of either human or rat S100A4 DNA can induce a metastatic phenotype upon nonmetastatic rat mammary tumour cells (Davies *et al*, 1993; Grigorian *et al*, 1993), whereas inhibition of S100A4 expression in metastatic cells can revert cells to a less metastatic phenotype (Maelandsmo *et al*, 1996). Notably, S100A4 is not tumorigenic *per se* as evidenced by the failure of transgenic mice overexpressing S100A4 to develop tumours (Ambartsumian *et al*, 1996; Davies *et al*, 1996), but instead acts as a potential inducer of metastasis in a given tumorigenic background. Transgenic mice generated with the S100A4 gene placed under the regulation of the mouse mammary tumour virus promoter leading to overexpression of S100A4 in lactating mammary glands yields transgenic animals that are phenotypically normal. However, crossing these transgenic mice with the GRS/A strain of mice (a strain characterised by a high incidence of mammary tumours that rarely metastasize) yielded a significant increase in mice that developed mammary tumours which metastasized (Ambartsumian *et al*, 1996). A similar result was obtained in mice bearing tumours expressing the HER2/Neu oncogene. It was found that the progeny from mice overexpressing S100A4 crossed with mice overexpressing the HER2/Neu oncogene develop tumours that metastasize more frequently and more rapidly than tumours in the parental neu mice (Davies *et al*, 1996). Interestingly, S100A4 knockout animals are able to develop spontaneous tumours at a late age and demonstrate reduction of apoptosis possibly as a result of a functional destabilisation of the tumour suppressor p53 (El-Naaman *et al*, 2004), a target protein of S100A4 (see below).

## PROPOSED FUNCTIONS FOR S100A4

Studies to determine the mechanistic basis for S100A4 function have shown a potential role for S100A4 in several different facets of tumour progression including motility, invasion, and apoptosis (Kriajevska *et al*, 1994; Takenaga *et al*, 1994a). Since S100A4 has no known enzymatic activity, interactions with other proteins, both intracellularly and extracellularly, are undoubtedly functionally critical. The interaction between S100A4 and its known protein partners has been shown to depend on both binding calcium through two EF-hand motif and dimerization. It has been proposed that S100A4 affects motility through its interaction with a nonmuscle myosin, a proposal strengthened by the colocalisation of S100A4 with myosin at the leading edge of motile cells (Kim and Helfman, 2003). Deletion analysis localised the interaction of S100A4 to the nonhelical C-term end of myosin heavy chain, specifically residues 1909–1937 of myosin heavy chain type Il-A, a region of MHC-IIA that includes a PKC phosphorylated residue, Ser 1917 (Kriajevska *et al*, 1998). Including S100A4 in an *in vitro* PKC kinase assay decreases the phosphorylation of MHC-IIA proportional to increasing amounts of S100A4 (Kriajevska *et al*, 1998). Despite the lack of a known functional role for the phosphorylation of myosin at Ser 1917, S100A4 decreases sedimentation suggesting that S100A4 may inhibit myosin filament assembly. Murakami *et al* (2000) reproduced these data using a 46 kDa C-term fragment of MHC-IIA, while also demonstrating a similar effect on MHC-IIB upon phosphorylation by PKC. Since PKC phosphorylation of MHC-IIA has no such effect on filament assembly, they proposed that S100A4 and PKC phosphorylation play parallel roles in the inhibition of polymerization of MHC-IIA and IIB, respectively. Interestingly, another one of the reported S100A4 protein partners, liprin *β*1 is prevented from being phosphorylated by the same two protein kinases, PKC and CK2 in the presence of S100A4 as well (Kriajevska *et al*, 2002). The tumour suppressor protein p53 has also been identified as a target for the S100A4 protein. S100A4 binds to the extreme end of the C-terminal regulatory domain of p53 *in vitro* and inhibits phosphorylation of the p53 C-terminal peptide by PKC but not by CKII. Activation of S100A4 expression in cell lines expressing wild-type p53 modulates transcription of p53 target genes (p21/WAF, bax, thrombospondin-1 and mdm-2). Interaction of the S100A4 with p53 tumour suppressor protein has been reported to induce the apoptosis promoting function of p53 (Grigorian *et al*, 2001).

S100A4 has also been reported to interact *in vitro* with actin filaments (Watanabe *et al*, 1993), with nonmuscle tropomyosin (Takenaga *et al*, 1994b) and possibly interacts with tubulin (Lakshmi *et al*, 1993). These data further suggest the possible involvement of S100A4 in the regulation of cell motility and cytoskeleton rearrangements. While interaction with the above-mentioned proteins has been documented to be calcium-dependent, there are reports of calcium-independent interactions of S100A4. Chen *et al* (2001) using an optical biosensor, demonstrated interaction of S100A4 with nonmuscle myosin and p53 in calcium-independent manner suggesting appearance of novel binding sites. Moreover, immunofluorescence studies revealed a novel, calcium-independent localisation of S100A4 to the leading edge of motile breast carcinoma cells (Kim and Helfman, 2003). Further studies will be required to determine the biological significance of the calcium-independent interactions and presumably its effectors.

It was also shown that S100A4 can interact with another member of S100 family. S100A4 can form heterodimers with S100A1 *in vitro* (Tarabykina *et al*, 2000; Wang *et al*, 2000). Significantly it was shown that the interaction between these two proteins, reduces motility and metastatic ability of S100A4 transformed cells (Wang *et al*, 2005).

## EXTRACELLULAR S100A4

S100A4 can also be secreted and once extracellular can affect several facets of tumour progression such as angiogenesis, stimulation of cell motility, upregulation of matrix metalloproteinases (MMPs), modulation of tumour-related transcription factors, and potentially as a stromal factor (Ambartsumian *et al*, 2001; Belot *et al*, 2002; Schmidt-Hansen *et al*, 2004a, 2004b). S100A4 may be released from both tumour and/or stroma cells by an as yet undetermined mechanism. S100A4 does not have any of the known signal sequences to target it for secretion through a classic vesicle transport pathway. Instead, it is hypothesized that S100A4 is released from the cell through an atypical pathway.

Proangiogenic capacity of S100A4 *in vivo* and its influence on endothelial cells' motility *in vitro* was the first indication of the extracellular significance of S100A4 in tumour progression (Ambartsumian *et al*, 2001). Angiogenic function of S100A4 was associated with its ability to upregulate the expression of several MMPs and downregulate their inhibitors (TIMPs) (Schmidt-Hansen *et al*, 2004b). Inhibition of S100A4 in tumour cells results in the downregulation of their invasive properties and reduces the expression of MMPs and TIMPs (Bjornland *et al*, 1999). Recombinant S100A4 stimulates motility and invasive growth of 3D capillary-like structures of mouse endothelial cells in Matrigel. The growth of the capillary structures is not dependent on stimulation of endothelial cells proliferation, but rather correlates with the transcriptional modulation of genes involved in cell motility and the proteolytic degradation of ECM. S100A4 activates transcription of MMP-11, MMP-13, MMP-14, and uPA in endothelial cells with the most pronounced increase in the MMP-13 gene. There was a concomitant transcriptional downregulation of two out of three genes coding for protease inhibitors – TIMP-l and TIMP-3, as well as PAT-I (Schmidt-Hansen *et al*, 2004b). More importantly, S100A4 induction of endothelial cells was accompanied by increased levels of MMP activity as demonstrated by casein zymography.

A significant impact of extracellular S100A4 protein was also demonstrated with S100A4-negative VMR cells whose metastatic capacity was strongly dependent on stromal interactions. Coinjection of these cells and S100A4 into the tail vein of mice followed by several administrations of S100A4 into the blood stream resulted in enhanced metastatic colonisation of the lung and liver. These data suggest that S100A4 may mimic stromal signals to activate tumour metastasis (Schmidt-Hansen *et al*, 2004a). Furthermore, VMR tumour cells release factor(s) that triggers a release of S100A4 from immortalised fibroblasts. In turn, extracellular S100A4 protein induces the remodelling of tumour cell cytoskeleton and adhesions contacts, stabilises the p53 protein and modulates expression of p53-regulated genes, as well as activating MMPs (Schmidt-Hansen *et al*, 2004b).

Expression of S100A4 protein in different types of tumours may suggest the ability of this protein to promote invasiveness and metastasis of various human neoplasms. However the actual mechanism of tumour-promoting function of S100A4 remains to be determined. Recent studies of an S100A4-deficient mouse model provided *in vivo* evidence that expression and release of S100A4 from activated stroma cells in tumour are central both for stimulation of tumour development and metastasis formation. When highly metastatic mouse mammary carcinoma cell line expressing high levels of S100A4 protein was introduced into S100A4-deficient mice, a severe impairment both in tumour incidence and uptake was observed. Moreover, no metastases were detected in the lungs of the S100A4(−/−), that finally grew tumours. This indicates that expression of S100A4 only in tumour cells is not sufficient for stimulation of tumour growth and metastasis, and implicates S100A4 expressing host-derived stroma cells as the main source of metastasis-stimulating activity of S100A4 (Grum-Schwensen *et al*, 2005).

Extracellular S100A4 was recently shown to influence cell survival in neuronal cells as well as neurite outgrowth. Addition of S100A4 resulted in protection of cells from the proapoptotic stimuli as detected by inhibition of DNA fragmentation but not caspases 3 and 6 activity (Pedersen *et al*, 2004). This cell protecting function of S100A4 may be beneficial for metastatic cells for their survival in blood stream and foreign microenvimnment of distal organs. However, an opposing scenario of response to S100A4 was recently reported by Pedersen *et al* (2004), who found that the S100A4-secreting human osteosarcoma cell line OHS are more sensitive to IFN-γ-mediated apoptosis than anti-S100A4 ribozyme transfected counterpart II-11b cells, which do not secrete S100A4 into conditioned medium. Addition of S100A4 to the cell medium of II-11b cells induces apoptosis. This discrepancy in response to extracellular S100A4 is suggestive of a strong dependence on cell type. Notably, recombinant S100A4 protein added to cell culture medium strongly stimulates differentiation of the rat hippocampal neurons (Novitskaya *et al*, 2000).

It is not presently known whether the biological activity of extracellular S100A4 is mediated via binding to a cell surface receptor and the triggering of signal transduction in responsive cells. However, the short exposure time (1–2 min) needed to generate the response of neurons to S100A4 and the fact that immobilised S100A4 protein can induce neuritogenesis indicates a putative receptor-mediated signalling. Indeed, it was shown that S100A4 treatment of hippocampal neurons induces activation signalling pathways similar to pathways that can be mediated by receptor tyrosine kinase, G protein-coupled receptors, or cytokine receptors (Novitskaya *et al*, 2000).

The receptor for advanced glycation end products (RAGE) has been identified as a putative receptor for several S100 proteins (Hofmann *et al*, 1999; Huttunen *et al*, 2000). Soluble peptide of RAGE blocks intracellular translocation of S100A4, S100A12, S100A13, and S100B in endothelial cells. The S100 proteins translocation was accompanied by activation of NF-κB, suggesting interactions between RAGE and the S100 proteins (Hsieh *et al*, 2004). The interaction between S100A4 and RAGE nevertheless remains inconclusive. Belot *et al* (2002) demonstrated that adding S100A4 to the culture medium of two separate astrocytic tumour cell lines strongly enhanced the migration rate of both cell lines and activated changes in actin cytoskeleton. However, RAGE mRNAs was found only in one of the cell line suggesting that the S100A4 effect on astrocytic tumour cell motility was not solely RAGE-mediated (Belot *et al*, 2002).

Taken together, we may speculate that S100A4 is an active metastasis regulator that is secreted by tumour cells and tumour-activated stromal cells. Extracellular S100A4 targets tumour or endothelial cells by binding to an as yet unknown receptor and triggering a signal transduction cascade and/or perhaps by internalisation followed by interaction with the intracellular target proteins. As a result, tumour cells acquire a more metastatic phenotype with altered cell adhesion, motility, high proteolytic activity, and increased survival potential.

## FUTURE PERSPECTIVES

There are many questions that remain about the cellular function of S100A4 and how it carries out its apparent influence on metastatic progression. Although S100A4 is known to bind to a number of cellular proteins, it remains to be determined how these interactions affect the function of the binding partners, and whether these interactions are relevant to the metastatic phenotype. Whether S100A4 alters oncogenic activation of specific signalling pathways leading to changes in differential expression of proteins needs to be explored. Discovering the function of a protein highly correlated with metastasis and poor prognosis may provide a leap forward in the treatment strategy of early cancer. By identifying and characterising the interaction of S100A4 with its effectors, we can supplement our understanding of how this particular clinical marker mechanistically engages cells to adopt a more motile, and thereby, more metastatic phenotype. Ultimately, this greater understanding will ideally lead to the use of S100A4 as not only a diagnostic and prognostic marker but also as a target for therapeutic design.
